# Supplementary material for: Effect of Familial Longevity on Frailty and Sarcopenia: A Case–Control Study
Source: Int J Environ Res Public Health. 2023 Jan 14;20(2):1534. doi: 10.3390/ijerph20021534 (PMC9865421; doi:10.3390/ijerph20021534)

Supplemental Material

Table S1: Comorbidities, n (%)

	Overall n = 176	Controls n = 88	Cases n = 88	p-value[a]
Arterial hypertension	102 (58)	55 (62.5)	47 (53.4)	0.256
Dyslipidemia	76 (43.2)	38 (43.2)	38 (43.2)	1
Fractures	64 (36.4)	37 (42)	27 (30.7)	0.212
Wrist	8 (4.5)	6 (6.8)	2 (2.3)	0.289
Vertebral	1 (0.6)	0	1 (1.1)	1
Hip	1 (0.6)	1 (1.1)	0	1
Others	54 (30.7)	30 (34.1)	24 (27.3)	0.429
Osteoarthritis	49 (27.8)	28 (31.8)	21 (23.9)	0.324
Cataracts	43 (24.4)	27 (30.7)	16 (18.2)	0.054
Prostatic hyperplasia[b]	16 (22.9)	10 (28.6)	6 (17.1)	0.344
Diabetes mellitus	37 (21)	20 (22.7)	17 (19.3)	0.700
Glaucoma	13 (7.4)	5 (5.7)	8 (9.1)	0.581
Asthma/COPD	11 (6.3)	7 (7.9)	4 (4.5)	0.453
Hypothyroidism	8 (4.5)	6 (6.8)	2 (2.3)	0.289
Heart failure	7 (4)	6 (6.8)	1 (1.1)	0.125
Gastroduodenal ulcer	7 (4)	4 (4.5)	3 (3.4)	1
Prostate cancer[b]	2 (2.9)	0	2 (5.7)	1
CAD	5 (2.8)	3 (3.4)	2 (2.3)	1
Breast cancer[c]	2 (1.9)	0	2 (3.8)	0.500
Parkinson	2 (1.1)	0	2 (2.3)	0.500
Uterus cancer[c]	1 (0.9)	1 (1.9)	0	1
Epilepsy	1 (0.6)	1 (1.1)	0	1
Hyperthyroidism	1 (0.6)	0	1 (1.1)	1
Chronic kidney disease	1 (0.6)	1 (1.1)	0	1
Colorectal cancer	0	0	0	-
Dementia	0	0	0	-
Lung cáncer	0	0	0	-
PAD	0	0	0	-
Stroke	0	0	0	-

Notes: n (%) = all variables in the table are presented with number (n) and percentaje (%) if n>0; COPD = chronic obstructive pulmonary disease; CAD = coronary artery disease; PAD = peripheral artery disease; [a] = McNemar test; [b] = percentage within men; [c] = percentage within women.

Table S2: Geriatric syndromes

	Overall n = 176	Controls n = 88	Cases n = 88	p-value[a]
Immobility, n (%)	1 (0.6)	0	1 (1.1)	1
Bowel control[b], n (%)	1 (0.6)	1 (1.1)	0	1
Bladder control[b], n (%)	2 (1.2)	1 (1.1)	1 (1.1)	1
Cognitive impairment[c], n (%)	3 (1.7)	2 (2.3)	1 (1.1)	0.500
Delirium, n (%)	0	0	0	-
Anxiety, n (%)	33 (18.8)	21 (23.9)	12 (13.6)	0.137
Depression, n (%)	38 (21.6)	18 (20.5)	20 (22.7)	0.850
Insomnia, n (%)	59 (34.9)	37 (44.6)	22 (25.6)	0.030*
Falls 12 months, n (%)	34 (21.1)	18 (22.2)	16 (19.8)	0.148
Falls 6 months, n (%)	22 (13.8)	12 (15)	10 (12.5)	1
Falls 3 months, n (%)	14 (8.7)	9 (11.1)	5 (6.3)	0.424
Hearing impairment, n (%)	15 (8.5)	7 (8)	8 (9.1)	1
Visual impairment, n (%)	26 (14.8)	17 (19.3)	9 (10.2)	0.134
No nutritional risk, n (%)	132 (75)	65 (73.9)	67 (76.1)	0.819
Nutritional risk, n (%)	44 (25)	23 (26.1)	21 (23.8)	0.819
High nutritional risk, n (%)	4 (2.3)	3 (3.4)	1 (1.1)	0.250
Polypharmacy, n (%)	67 (38.1)	43 (48.9)	24 (27.3)	0.003*
Pain[d], n (%)	61 (34.7)	38 (43.2)	23 (26.1)	0.029*
Days/week, median (IQR)	5 (4)	6 (3)	4.5 (4)	0.581
VAS, mean ± SD	5.2 ± 2.1	5.5 ± 2.1	4.7 ±1.9	0.402
Constipation, n (%)	35 (19.9)	16 (18.2)	19 (21.6)	0.571
Dysphagia, n (%)	0	0	0	-
Pressure ulcers, n (%)	0	0	0	-

Notes: n (%) = number and percentage for categorical variables if n>0; IQR= interquartile range; SD = standard deviation; VAS = visual analog scale; * = p<0.05; [a] = McNemar test for categorical variables, Student's t-test for related samples for quantitative variables with normal distribution, Wilcoxon signed rank test for matched samples for non-normal quantitative variables; [b] = occasional incontinence; [c] = age and education adjusted; [d] = 2 days per week or more;

Table S3: Pearson correlations among total, LDL cholesterol and Body mass index

Overall Sample			
	Total cholesterol	Coefficient	-0.203
		p-value	0.007
	LDL-cholesterol	Coefficient	-0.159
		p-value	0.036
Case-group			
	Total cholesterol	Coefficient	-0.098
		p-value	0.364
	LDL-cholesterol	Coefficient	-0.113
		p-value	0.298
Control-group			
	Total cholesterol	Coefficient	-0.241
		p-value	0.024
	LDL-cholesterol	Coefficient	-0.148
		p-value	0,169

Table S4: Body mass index, IL-6 and frailty

	Robusts	Prefrail	Frail	p- value*
	% within group			
**BMI**				0.346
Underweight	0.0	80.0	20.0	
Normal weight	23.3	60.0	16.7	
Overweight	30.4	57.0	12.7	
Obese	16.4	65.6	18.0	
	mean (Standard deviation)			
**IL-6**	1.33 (1.13)	1.41 (0.91)	1.60 (0.84)	0.185

Notes: * Chi Square for BMI and Kruskal Wallis for Interleukin-6 (IL-6)

Table S5: Lifestyle habits and frailty

	Robusts	Prefrail	Frail	p- value*
	% within group			
**Current Smoker**				0.168
No	22.9	63.4	13.7	
Yes	30.4	43.5	26.1	
**Alcohol**				0.481
No	24.4	58.3	17.3	
Yes	21.7	67.4	10.9	
**Physical exercise**				0.174
No	18.9	58.5	22.6	
Yes	26.0	61.8	12.2	
**Physical activity**				0.000
Mild	12.5	65.6	21.9	
Moderate or vigorous	37.3	54.7	8.0	

Notes: * Chi Square

Table S6: Sarcopenia patients according to their BMI

BMI	Sarcopenia		Cases	Controls
	N	%	N	%
Underweight	2	18,2	1 (16.7)	1 (20)
Normal weight	7	63,6	4 (66.7)	3 (60)
Overweight	2	18,2	1 (16.7)	1 (20)
Obese	0	0	0	0
Total	11	100	6	5

Notes: N = number of patients. % = percentage. BMI = body mass index

Table S7: Body mass index, Ldl and total cholesterol

	Obese	Mean	Standard deviation	p-value
LDL-cholesterol	No	118,5	35,9	0,163
	Yes	110,7	34,1	
Total cholesterol	No	201,9	39,6	0,197
	Yes	193,8	39,4	
	**Overweight**			
LDL-cholesterol	No	112,4	34,2	0,163
	Yes	119,9	36,5	
Total cholesterol	No	197,8	39,4	0,618
	Yes	200,8	40,0	
	**Normal weight**			
LDL-cholesterol	No	116,7	36,7	0,484
	Yes	111,7	28,4	
Total cholesterol	No	198,7	40,4	0,767
	Yes	201,1	35,9	
	**Underweihght**			
LDL-cholesterol	No	115,2	34,4	0,159
	Yes	137,8	60,6	
Total cholesterol	No	198,3	39,0	0,126
	Yes	225,8	54,4	

Figure S1. Charlson index; Differences between matched case-control pairs.
Y axis: percentaje of pairs.
X axis: Result of the substraction of Charlson of the case minus Charlson of the control
Red columns: Percentaje of pairs whose cases have less Charlson Index than controls
Grey columns: Percentage of pairs with no differences
Blue columns: Percentaje of pairs whose cases have higher Charlson Index than controls
Significance with Wilcoxon matched pair test.
Panel A = Overall sample.
Panel B = Over 70 years old pairs.

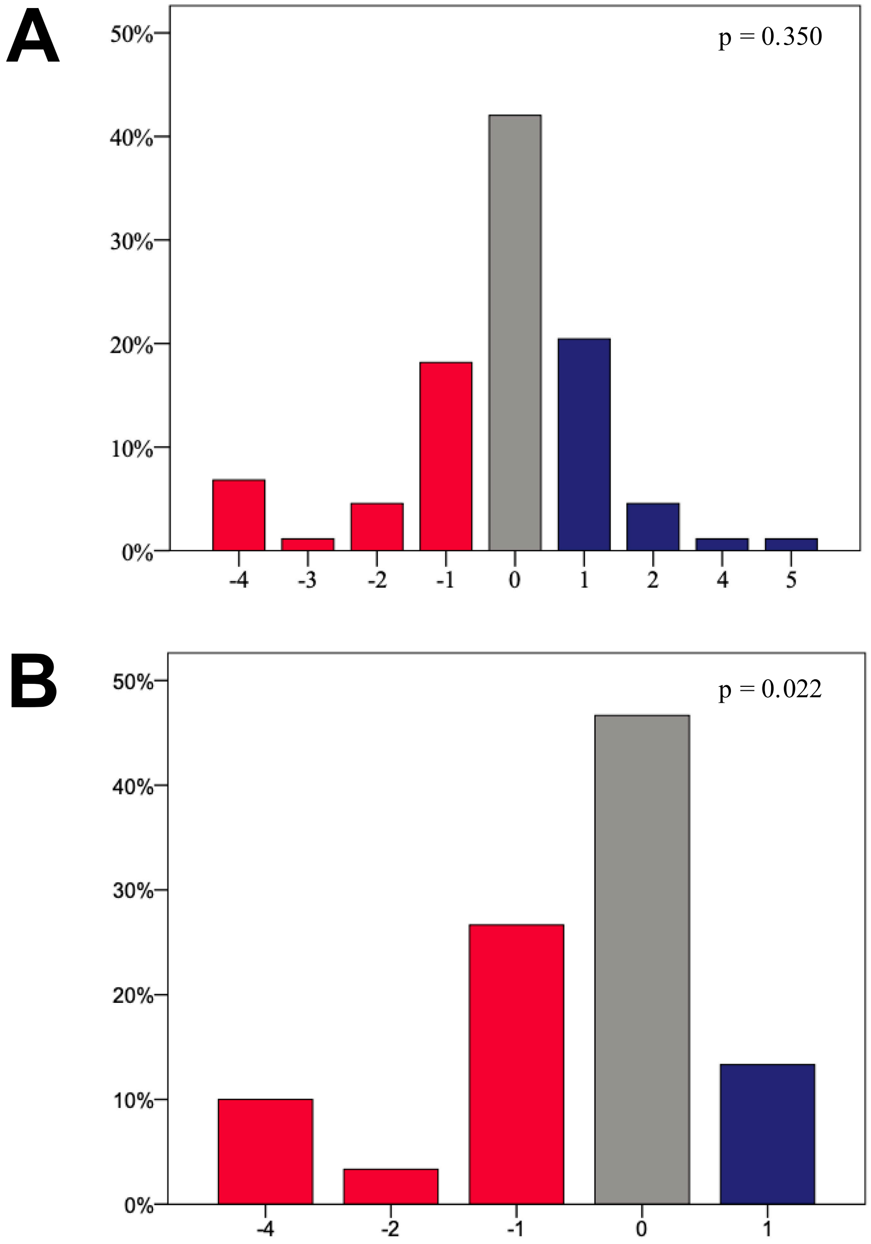

Figure S2. Charlson index.
Y axis: Mean of Charlson index.
X axis: Cases (red). Controls (blue).
Error bars represent: 95% confidence intervals.
Panel A = Overall sample.
Panel B = Over 70 years old pairs.

A

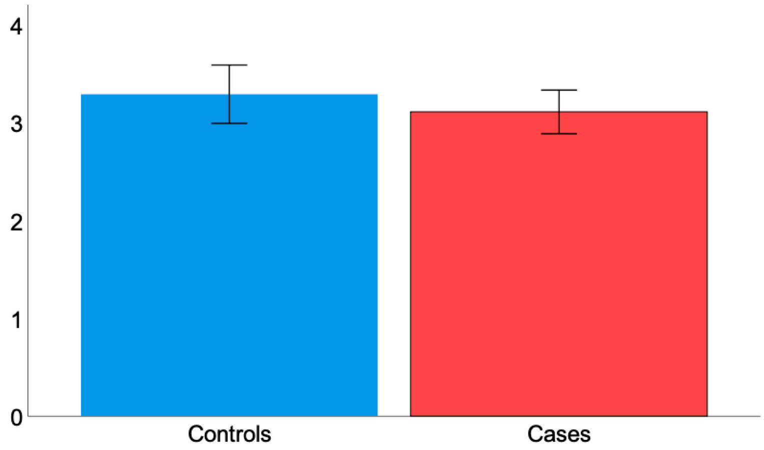

B

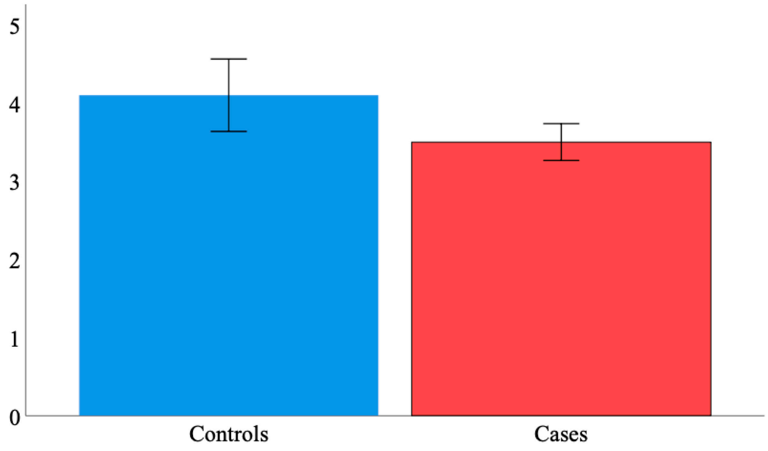

Supplement: Supplementary file 1 [file ijerph-20-01534-s001.zip › ijerph-2095071-supplementary.pdf]
